# Using Qualitative Methods to Validate and Contextualize Quantitative Findings: A Case Study of Research on Sexual Behavior and Gender-Based Violence Among Young Swazi Women

**DOI:** 10.9745/GHSP-D-16-00062

**Published:** 2016-09-28

**Authors:** Allison Ruark, Rebecca Fielding-Miller

**Affiliations:** a Brown University, Department of Medicine, Providence, RI, USA; b University of California, San Diego, Division of Global Public Health, San Diego, CA, USA

## Abstract

Nesting qualitative data collection methods within quantitative studies improves results by assessing validity and providing depth and context. Using data from 3 sources from Swaziland, we triangulate qualitative and quantitative findings to highlight how different methodologies produce discrepant data regarding risky sexual behaviors among young women. We found that women reported similar numbers of lifetime sex partners in all sources, but the proportion reporting multiple and concurrent partnerships was several times higher in qualitative interviews. In addition, qualitative data can provide deeper understanding of how participants, such as those experiencing gender-based violence, understood the experiences behind the quantitative statistics.

*Dr. Allison Ruark and Dr. Rebecca Fielding-Miller contributed equally to the writing of this article*.

## INTRODUCTION

Most modern public health researchers in the behavioral and social sciences situate their research within a post-positivist framework, either explicitly or implicitly.^1^^,^^2^ Researchers working within a post-positivist framework assume that while objective “truths” of human behavior and experience exist, measuring and defining these realities is at best an approximate science. A physician or clinical researcher can measure blood pressure or CD4 count using precisely calibrated instruments and feel confident in the accuracy of the measurements, but quantifying aspects of human health and well-being is not so simple.

Measuring and defining human behavior and experience is at best an approximate science.

Social scientists and public health practitioners face multiple challenges in determining how best to measure social phenomena and various behaviors relevant to public health and how to define precisely what to measure. Efforts to conceptualize and assess important constructs such as self-efficacy, stigma, social norms, sexual identity, violence, and sexual behavior have generated a great deal of research and debate.^3^^-^^7^ From a post-positivist point of view, these phenomena are subjective by their very nature, making them impossible to precisely define and measure, especially in a way that is meaningful across all contexts.

In this commentary, we consider the challenges of collecting and interpreting data on sexual behavior and gender-based violence (GBV). We present a case study that illustrates challenges and potential solutions to maximize data validity and describe these behaviors and experiences as closely as possible. The comparisons and concepts come from our experience conducting 2 separate research studies in Swaziland in 2013–2014, both of which characterized sexual behavior among Swazi women in their 20s and 30s. We did not set out to collect comparable data, but we noticed that our research studies (1 qualitative and 1 quantitative using audio computer-assisted self-interviewing [ACASI]) produced very different findings about sexual behavior in research populations that seemed to be quite similar. Our data also differed markedly from the latest Swaziland Demographic and Health Survey (DHS).^8^ These observations led to further consideration of how different data collection methodologies and various sources of bias may influence the story that participants tell in a research interview, and how closely our research findings reflect the “true” nature of behaviors in our study populations. We believe that frank consideration of the strengths and weaknesses of data collection methodologies is critical to maximizing the validity of collected data and the value of research.

Frank consideration of the strengths and weaknesses of data collection methodologies is critical to maximizing the validity of collected data and the value of research.

## COLLECTION OF BEHAVIORAL DATA

The collection of behavioral data in public health research rests on 2 assumptions. First, that research participants have life experiences and engage in behaviors that influence their health risks and outcomes. Second, that data collected through behavioral research can measure the “true” nature of these experiences and behaviors with enough accuracy to be useful to interventions designed to mitigate health risks. The challenge of behavioral research is to minimize the degree of error and bias, which is inevitable in all research studies, but especially in studies that use self-reported behaviors on sensitive topics.

Collecting data through self-report is often necessary for sensitive topics such as sexual history, experience or perpetration of violence, or other phenomena for which observation is problematic or impossible. Self-reported data on sensitive topics are subject to a number of well-recognized potential biases, including social desirability bias, item response bias, reporting bias, and recall bias.^9^ People may report their sexual behavior inconsistently over time,^10^ and self-reports of sexual behavior have been found to be inconsistent with biological data^11^ and reports of sexual partners.^12^ Many types of bias derive from the data collection activity itself and are influenced by the methodology used and the skill and identity of the data collector.

There are many reasons why research participants may choose to represent their stories in research settings in a certain way.^2^ The interview is a “situated, social activity” in which the person being interviewed “produces, reproduces, and articulates” an identity, largely in response to rapport with and perceptions of the interviewer,^13^ and in response to “situational, cognitive, social, and psychological factors”.^14^ While there is likely no research methodology that can consistently deliver data that perfectly represent reality, there are many good reasons to strive to improve the validity of the data we collect. For example, using qualitative data collection methods to understand sexual risks and experiences of sexual violence in a population can result in better interventions.

Using qualitative methods to collect data on sensitive topics, such as sexual history or violence, can provide critical insight and context, resulting in better interventions.

### Comparing the Validity of Different Methods of Collecting Data on Sexual Behavior and Gender-Based Violence

Various studies provide evidence that study interviewers can influence participants’ reports of sexual behavior^15^^,^^16^ or experiences of violence.^17^^,^^18^ In South Africa, respondents reported more conservative sexual behavior (fewer lifetime sexual partners and more condom use) to older interviewers, and men were especially likely to report fewer lifetime sexual partners to male interviewers.^19^ In Uganda, women were more likely to report sexual activity and willingness to use condoms to male interviewers compared with female interviewers.^20^ In Malawi, adolescent girls were more likely to report having had sex when asked by a nurse before testing for sexually transmitted infections, compared with face-to-face interviews or ACASI.^10^ In qualitative research, the researcher is the instrument used to collect data,^21^ and rapport between the researcher and participant is a critical aspect of data collection.

In qualitative research, the researcher is the instrument used to collect data, and therefore rapport between the researcher and participant is a critical aspect.

ACASI has frequently been employed to increase confidentiality and data validity in research on sexual behavior. ACASI has generally been found to yield higher reports of some sensitive sexual behaviors, but not others, compared with face-to-face interviews,^15^^,^^16^^,^^22^^,^^23^ differing in some cases by respondent gender.^24^ Two 2010 reviews yielded somewhat different conclusions. Langhaug and colleagues concluded that there was “strong evidence” that computer-assisted interviewing increased reports of sensitive sexual behaviors in developing countries.^25^ Phillips and colleagues conducted a meta-analysis of data from low- and middle-income countries (LMICs). They concluded that compared with face-to-face interviews, other methods did not consistently yield higher reports of ever having sex, non-condom use, or number of sexual partners, but did produce higher reports of forced sex.^26^

We identified few studies that compared the validity of various methodologies for collecting self-reported data on GBV experiences. Interviewer training and skill is likely an important factor, and without specific training on the nature of GBV and sexual assault, even highly trained research assistants may struggle with how to categorize a particular event.^27^ Evaluations from the United States and Canada suggest that ACASI may capture more reports of intimate partner violence than face-to-face interviews,^28^ and that many women prefer disclosing these experiences to a computer rather than to another person.^29^ However, very little evidence exists on the relative validity of using ACASI to measure self-reports of violence in LMICs. In low-income communities in Bangalore, India, young married women reported significantly *fewer* experiences of domestic violence via ACASI than they did in face-to-face interviews.^30^ In these contexts, face-to-face interviews may have had higher disclosure because of their perceived cathartic value, or because of the potential of being connected to services in otherwise low-resource settings.^30^

The value of combining quantitative and qualitative methods in research of violence has been recognized, but few studies using this approach in lower- and middle-income countries were found in the literature.

Qualitative methods can increase opportunities for building trust between an interviewer and a participant and contain the flexibility to enable the participant to co-construct the interview and introduce new topics of inquiry.^21^ These attributes of qualitative research may produce data that are richer, more nuanced, and more valid than data collected through quantitative means. Studies of individual sexual behavior and sexual violence typically use in-depth interviews (IDIs) rather than focus group discussions (FGDs). The privacy and confidentiality of IDIs encourages participants to share their personal opinions on sensitive topics, whereas FGDs are more likely to capture data on community norms, or what FGD participants believe is socially acceptable to say in front of others.^21^^,^^31^^,^^32^

Survivors of violence may construct their experiences in a variety of ways depending on their cultural context, current life circumstances, and the interview scenario itself, and therefore GBV studies may particularly benefit from a qualitative approach that allows space for nuance and flexibility.^33^ Survivors may be reluctant to discuss experiences of violence out of shame, particularly with survey interviewers with whom they have little rapport.^17^^,^^18^ Survivors may not recall their experiences, or they may reconstruct what occurred in a way that distances them from stigmatized identities (such as that of a rape or sexual assault victim).^18^ A qualitative exploration in South Africa found that women who described non-consensual, coerced, or violent sexual experiences with intimate partners would frequently describe these experiences as disappointing, emotionally hurtful, or traumatic, but rarely categorized them as rape and often attributed them to men’s “natural” sexual drives and entitlement.^34^

GBV studies may particularly benefit from a qualitative approach that allows space for nuance and flexibility.

### The Importance of Using Mixed Methods in Research on Sexual Behavior

It is not uncommon for large research trials to use qualitative data to contextualize quantitative findings about sexual behavior.^35^^-^^39^ While the value of combining quantitative and qualitative methods in research of violence has been recognized,^27^ few studies using such a mixed-method approach in LMIC contexts were found in the literature. Schatz and Williams note that many researchers have called for mixed-methods research on topics related to gender, and issue a specific call for qualitative studies to validate and contextualize DHS data on gender inequality.^40^ Qualitative research can inform the development of structured, quantitative questionnaires,^41^ establish which words or phrases are locally understood to refer to acts of violence such as rape or coerced sex,^42^ or aid researchers in navigating complex cultural minefields as they ask sensitive questions about sex and violence.^42^ Qualitative methods also provide context. For example, a study of GBV in the Democratic Republic of the Congo used FGDs with women who had survived violence to further explore topics addressed in a quantitative survey. The open-ended nature of the FGDs enabled women to voice concerns and priorities that had not been addressed in the quantitative survey instrument, resulting in suggestions for further research.^43^

Despite the frequency with which qualitative and quantitative methods are used in the same project, we identified only 1 study that used qualitative data to *validate* quantitative sexual behavior data. In Malawi, a qualitative study nested within a larger quantitative project found that more young women and men reported having ever had sex in IDIs compared with face-to-face surveys; 39% of young women and 17% of men gave discrepant answers in the 2 interview modalities.^13^ In an analysis of the IDIs, Poulin concluded that they allowed for “flexibility and reciprocal exchange” that did not exist in the surveys, thus producing trust between the interviewer and participant and resulting in more accurate reporting.^13^ Repeated IDIs may be especially effective in increasing rapport, and may also be useful for collecting longitudinal data on the experiences of participants over time.^35^^,^^44^^-^^46^

## CASE STUDY COMPARING DATA ON SEXUAL BEHAVIOR AND GBV FROM DIFFERENT SOURCES

In this case study, we compare data from 3 sources: the Swaziland DHS 2006–2007; a quantitative ACASI survey of young women’s sexual histories that included questions on GBV; and a qualitative interview-based study of young women’s sexual partnerships that also elicited data about GBV. We provide this case study as a practical example for public health researchers and practitioners who wish to integrate qualitative methods into a quantitative study. We believe this approach can lead to better research and outcomes.

### Swaziland Demographic and Health Survey

The 2006–2007 Swaziland DHS was a large, nationally representative survey carried out by the Swaziland Central Statistics Office in partnership with Macro International and the first and only DHS to be conducted in Swaziland.^8^ Trained Swazi data collectors administered face-to-face interviews with participants from all 4 regions of Swaziland between July 2006 and February 2007. The DHS report does not mention any effort to match interviewers to respondents by age or gender. The Woman’s Questionnaire took an average of 2 hours to complete and included questions about demographic characteristics and attitudes and behaviors related to fertility and health, including a sexual partner history extending to the 3 most recent sexual partners. Nearly 5,000 women ages 15 to 49 were included in the DHS (a 94% response rate). In this case study we present weighted data for 2,767 women ages 20 to 39 who reported ever having sex to increase comparability to other data presented. We had no role in collecting these data, but the first author (AR) extracted age-specific data from datasets made available at www.dhsprogram.com.

### Audio Computer-Assisted Self-Interview Survey

Between February and June 2014, the second author (RFM) conducted a quantitative survey with 406 pregnant women ages 18 to 42 accessing antenatal care in 1 rural and 1 urban public health clinic.^47^^,^^48^ In this case study, we present a sub-sample of 340 women ages 20 to 39. The survey was part of a larger mixed-methods project designed to operationalize and measure how Swazi women conceptualize transactional sex. The survey instrument included questions about lifetime and 12-month sexual partner history as well as questions adapted from the World Health Organization’s violence against women instrument based on previous work conducted in South Africa.^17^^,^^49^ After formative and qualitative research, the survey instrument was tested for face validity with content experts and Swazi colleagues, translated into siSwati, and back-translated into English to check translation accuracy. It was then piloted in siSwati using cognitive interviewing^50^ at an urban public health clinic.

Data collection was carried out using ACASI, with a young Swazi female research assistant narrating the audio track in siSwati. Participants were systematically sampled from women awaiting antenatal services at public clinics. A young female Swazi research assistant coached participants on the use of laptop computers for the initial set of demographic questions and then withdrew unless a participant requested assistance. The final survey took about 45 minutes to complete, with an approximately 54% response rate. Per the request of the Swaziland Scientific and Ethics Committee (SEC), no incentives were offered, which may have resulted in the low response rate. Participants were offered food, drink, and childcare. Further details are available elsewhere.^48^ The SEC and Emory University Institutional Review Board reviewed and approved the study protocol.

### Qualitative Interviews

From June 2013 to September 2014, the first author (AR) carried out a qualitative ethnographic study of the transitions and trajectories of young Swazi adults’ sexual partnerships.^51^^,^^52^ Data presented here are from repeated in-depth life-course interviews with 14 Swazi women between the ages of 20 and 39. Participants were recruited from a central location in Mbabane, the capital of Swaziland, and were purposively sampled to provide variation in education level, marital and relationship status, and place of residence (urban, peri-urban, and rural). Interviews were carried out in siSwati or in a mixture of English and siSwati by trained Swazi interviewers who were themselves young women in their 20s and 30s.

Each woman was interviewed 3 to 5 times, with the total average interview time per woman being over 3 hours and the average time between first and last interview being 9 months. Interviews were semi-structured and addressed family backgrounds and sexual partnership history, with emphasis given to the chronology and overlap of sexual partnerships. Each woman was encouraged to tell the story of each of her sexual partnerships in as much detail as she was willing to divulge. Further details about the methodology of this study are provided elsewhere.^51^^,^^52^ The SEC and the Institutional Review Board of the Miriam Hospital (Providence, RI, USA) approved this study.

## USING QUALITATIVE DATA FOR TRIANGULATION: SEXUAL BEHAVIOR

**FIGURE 1.**
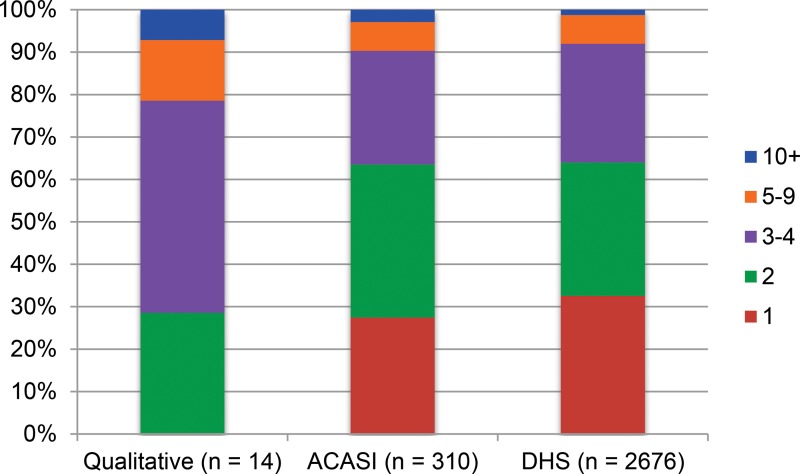
Number of Sexual Partners, Lifetime Abbreviations: ACASI, audio computer‐assisted self‐interviewing; DHS, Demographic and Health Survey.

**FIGURE 2.**
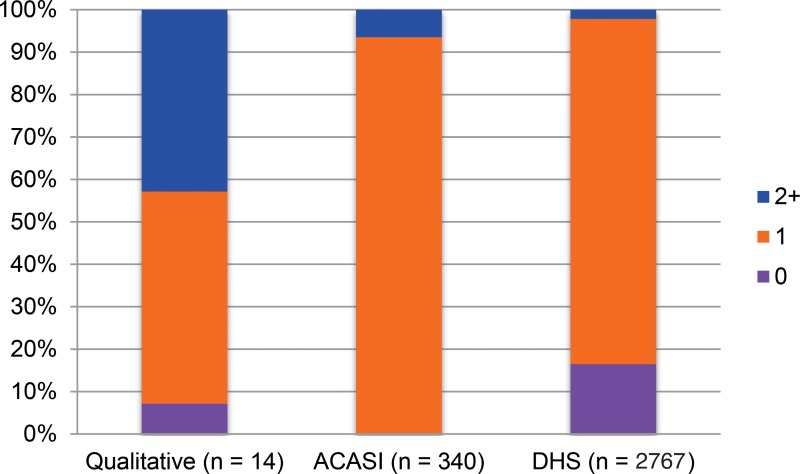
Number of Sexual Partners, Past 12 Months Abbreviations: ACASI, audio computer‐assisted self‐interviewing; DHS, Demographic and Health Survey.

**FIGURE 3.**
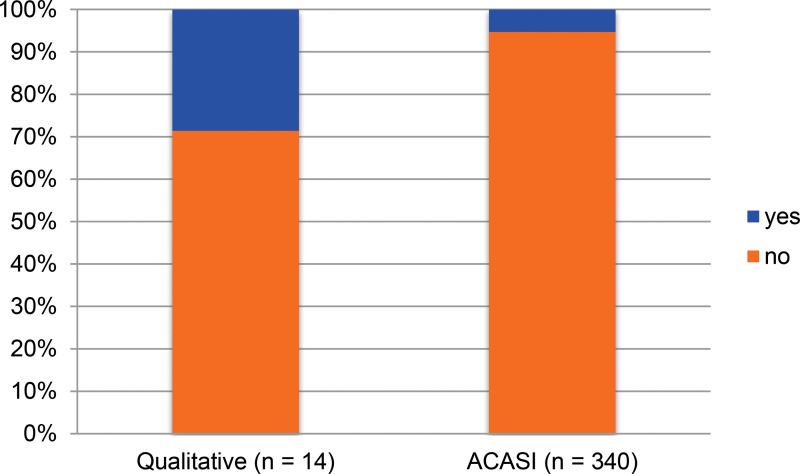
Concurrent Sexual Partners, Past 12 Months Abbreviation: ACASI, audio computer‐assisted self‐interviewing.

In Figure 1, Figure 2, and Figure 3, we present sexual history data derived from the 3 sources described. Triangulating research findings from different sources provides a validity check to all data sources. We noted that women reported similar numbers of lifetime sexual partners in all surveys, with a very similar proportion of participants reporting 1 or 2 lifetime sexual partners in ACASI and DHS data, and only a small minority in all 3 surveys reporting 5 or more lifetime sexual partners. The proportion of women reporting multiple and concurrent sexual partnerships in qualitative interviews was several times that observed in the quantitative surveys, however. A substantial minority of women reported only 1 lifetime sexual partner in both ACASI and DHS data, but no participants in the qualitative interviews did so. The proportion of women who reported 2 or more sexual partners in the past 12 months among qualitative interview participants was an order of magnitude greater than the proportion reporting multiple partners among ACASI and DHS participants. Similarly, participants in the qualitative interviews were several times more likely to report having concurrent partners in the past 12 months than were participants in the ACASI survey.

In all 3 data sources, women reported similar numbers of lifetime sexual partners, but the proportion of women reporting multiple and concurrent partnerships in qualitative interviews was several times that reported in quantitative surveys.

We do not argue that these data are directly comparable, and we have intentionally presented them visually rather than numerically so as *not* to invite statistical comparison. Calculating the magnitude and significance of differences between data from these discrepant sources would be epistemologically and statistically inappropriate. These data were drawn from different populations at different points over a decade, and with somewhat different inclusion criteria. By definition, all women participating in the ACASI study had reported at least 1 sexual partner in the preceding 12 months. The ACASI and qualitative studies also used convenience samples while the DHS data attempted to create a nationally representative sample.

All data describe young, sexually experienced women between the ages of 20 and 39 in Swaziland. We believe the observed differences between the 3 sources are striking and strongly suggest that qualitative methods may produce higher reports of sensitive sexual behaviors than do standard quantitative surveys. We assume that Swazi women will be highly unlikely to over*-*report socially stigmatized behaviors (such as multiple and concurrent sexual partnerships), and therefore that data showing higher levels of socially stigmatized behaviors are more accurate.

The differences between the 3 sources strongly suggest that qualitative methods may produce higher reports of sensitive sexual behaviors than quantitative surveys.

The qualitative methods may have produced higher reports of multiple and concurrent sexual partners for 2 reasons. First, an in-depth interview enables a conversation between an interviewer and a participant that elicits a detailed story rather than isolated points of data, reducing the possibility of misunderstanding.^21^ The longitudinal and iterative nature of the research allowed interviewers to probe and confirm information over multiple interviews (in some cases gently challenging reports that seemed inconsistent or lacking in credibility), and to detect circumstances of risk (such as concurrent sexual partners) that may not have emerged in a once-off interview. Second, repeated interviews and the prolonged nature of the relationship between interviewer and participant created trust and rapport, which we believe increased participants’ willingness to reveal sensitive information. In many cases, additional interviews increased frankness and disclosure as interviewers built rapport with participants over time, resulting in reporting of additional sexual partners.

In-depth interviews elicit detailed stories that can reduce the possibility of misunderstanding, and repeated interviews can increase participants’ willingness to reveal sensitive information.

## USING QUALITATIVE DATA FOR CONTEXTUALIZATION

**FIGURE 4.**
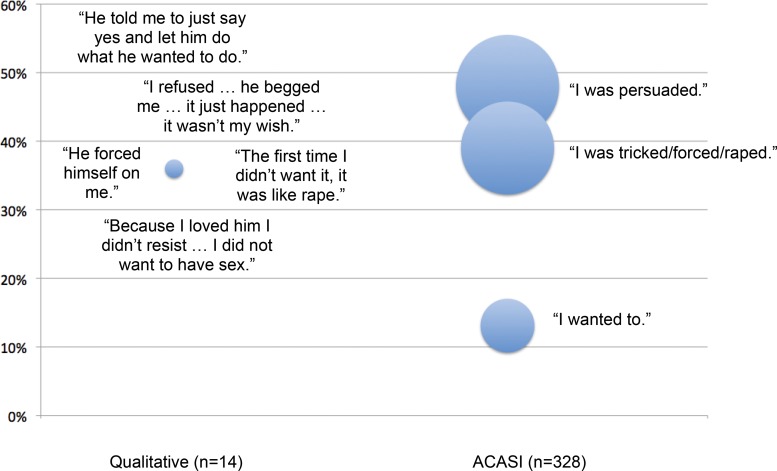
Experiences of Young Swazi Women During First Sex, Qualitative Research Findings Compared With Quantitative ACASI Study Findings Abbreviation: ACASI, audio computer‐assisted self‐interviewing. Each quote is plotted on the vertical axis according to the proportion of women who reported each outcome. For the qualitative data, 5 of 14 women reported coerced or forced first sex, and a single quote represents an individual woman’s account. The size of each bubble represents the absolute number of women reporting each outcome.

In the qualitative and ACASI studies, we asked women how they would describe their first sexual experience. The qualitative study focused on participants’ own interpretation of their experience, whereas the ACASI study asked them to select one of multiple preexisting options: “I wanted to,” “I was persuaded,” “I was tricked,” “I was forced”, or “I was raped.” A comparison of results is shown in Figure 4, with each bubble plotted on the vertical axis according to the proportion of women who reported each outcome, and with the size of each bubble representative of the absolute number of women. For the qualitative data, a brief quote is presented for each woman who reported coerced or forced first sex. While we deliberately present these as a diagram to discourage direct statistical comparisons that would be inconsistent with the nature of the data, we do note that just over one-third of women in both samples described their first sexual experience as forced or coerced. This suggests that a well-implemented ACASI survey can produce similar levels of disclosure as same-gender, face-to-face interviews with strong rapport—the suggested best practice for collecting data on violence against women.^17^^,^^53^

In addition to this validity check of the quantitative ACASI data, triangulating survey findings with qualitative data provides a deeper understanding of how participants understood the experiences behind the statistics, and how their understanding may have shifted over time. Just less than half of ACASI respondents reported acquiescing to sex after a partner “persuaded” or “begged” them (Figure 4). However, from the ACASI survey alone we do not know precisely how women may have experienced an encounter that they later labeled as “persuasion.” Restrictive cultural norms may lead participants to select the “persuaded” option to describe a fully consensual and enthusiastic encounter if they feel it is culturally unacceptable for women to express strong sexual desire.^54^ Conversely, women who reported being “persuaded” by a partner could also be revising traumatic events because they feel shame admitting to experiences of sexual violence. In the qualitative data, some women recast violent or coercive events into acts of love or desire, such as this account of a woman’s first sexual partner:

He used to overpower me, to be honest. We didn’t have sex because we were in love … He took advantage of me and I could see that he wanted to have sex with me and I refused. He said that I couldn’t refuse now and he carried on … I got used to him even though I was scared of him … He saw that so he tried to bring me closer by apologizing and the relationship was okay from there.

Triangulating quantitative findings with qualitative data provides a deeper understanding of how participants understood the experiences behind the statistics, and how their understanding may have shifted over time.

While this participant might choose the category “I was persuaded,” given limited response options in a quantitative survey about her first sexual encounter, her account suggests the difficulty of subsuming complex experiences into a single descriptor or category. We suggest there is a need for nested qualitative research to build context and “thick” description^55^—rich, contextualized description of human behavior—into larger observational and survey-style studies on subjects such as GBV.

## RECOMMENDATIONS

Based on a comparison of multiple data sources from Swaziland, we suggest that qualitative methods have an important role to play in research studies, including surveillance, observational, and experimental studies. Formative qualitative work before and during a quantitative survey may identify potentially unclear questions and language, improving the quality of the survey questions and final interpretation of the data.^56^ We also recommend, whenever possible, nesting qualitative data collection within quantitative studies of sensitive topics such as sexual behavior and GBV, in a sequential explanatory design,^57^ which gives priority to quantitative data but uses qualitative data to provide validation and insight into the meaning of the quantitative data (contextualization).

We recommend nesting qualitative data collection within quantitative studies of sensitive topics such as sexual behavior and GBV.

### Validation

For topics that may benefit from better rapport between an interviewer and participant, and the opportunity to probe or revisit topics over the course of an interview, we recommend systematically sampling participants from the quantitative survey and inviting them to participate in a qualitative interview on the same topic. Although qualitative research is not traditionally used to generate statistics, data from a systematically sampled, representative subsample could provide a useful validation check on the larger quantitative project.

### Contextualization

For sensitive or ambiguous topics, in addition to rigorous qualitative formative work to build valid survey instruments, we recommend purposively sampling a subsection of participants who have participated in the quantitative data collection process, or from a similar population, to better understand the context and potential shifting meanings within a survey item. Both the meth-odology and underlying philosophy of qualitative research provide the flexibility to understand and report the sometimes ambiguous data that result as participants construct and reconstruct traumatic or sensitive experiences. The goal in this case is not to compare and contrast qualitative and quantitative findings, but rather to continue with qualitative investigation until the qualitative data have provided as rich, nuanced, and complete an understanding of the quantitative data as possible.

Purposively sampling a subsection of informants who participated in a quantitative data collection process can help researchers better understand context and potential shifting meanings.

### Rigor

The process of designing a qualitative study, or conducting qualitative interviews or focus groups, requires specific skill sets and explicit training. Acquiring credible, dependable, and confirmable^58^ qualitative data to complement quantitative data requires careful thought and an understanding of why and how a given qualitative method (i.e., IDIs, FGDs, observation) is best suited to the question. It is also critically important to select interviewers whose age, gender, social background, and life experiences enable them to create the right kind of rapport with interview participants. Qualitative interviewers require training specific to qualitative approaches and methods to help them build rapport with a participant, feel confident deviating from interview guides when appropriate, and probe deeply to draw out participant stories.

Qualitative interviewers require training specific to qualitative approaches and methods to successfully draw out participant stories.

## LIMITATIONS OF QUALITATIVE AND QUANTITATIVE METHODS

Despite the importance of qualitative methods, particularly IDIs, we note that they are not appropriate for all research objectives nor are they the panacea for all data quality issues. Qualitative methods are not intended to produce generalizable statistical inferences, and they are time and energy intensive, making qualitative studies with large numbers of participants impractical. The iterative nature of data collection and analysis is also inherently dependent on the researcher-as-instrument, requiring intense and specific training to assure data quality.^58^ As we discuss in this commentary, quantitative methods also have substantial weaknesses; they lack the flexibility and iterative approach of qualitative research and cannot detect or correct for the distance between what a participant reports and the “truth.” Mixed-method approaches have the potential to enable qualitative and quantitative methods to work together in complementary and synergistic ways, resulting in higher-quality research.

## CONCLUSION

In this commentary, we present a case study comparing 3 sources of data on sexual behavior and GBV experiences of young women in Swaziland. We highlight discrepant findings not for the purpose of statistical comparison, but as a means of discussing the importance of data collection methodology and the unique strengths of qualitative methods in providing validation and contextualization for quantitative data. The higher frequency of multiple and concurrent sexual partnerships and the rich description of GBV provided in the qualitative study suggest that qualitative methods may more closely approach the “truth” of certain behaviors and experiences. Our objective in this commentary is not to offer definitive answers regarding sexual behavior and GBV among young women in Swaziland, but to raise questions—and offer suggestions—about how research might better capture sensitive behaviors and experiences. We argue that qualitative methods are critical and underused in validating and contextualizing data collected through quantitative methods.

## Notes

**Peer Reviewed**
